# Prebiotic nucleic acids need space to grow

**DOI:** 10.1038/s41467-018-07221-x

**Published:** 2018-12-12

**Authors:** Daniel Whitaker, Matthew W. Powner

**Affiliations:** Department of Chemistry, University College London, 20 Gordon Street, London, WC1H 0AJ UK

## Abstract

What were the conditions on early Earth when nucleotides were formed, and what are the most plausible nucleoside candidates? Answering these questions will require mechanistic chemistry and planetary science to work together, enhancing not limiting each other’s scope of investigation.

## Introduction

RNA is the leading candidate for life’s first information-carrying biopolymer because of its dual role in information transfer and biocatalysis, as well as the deep-seated evolutionary history of non-coding RNAs. It is currently unknown when ribonucleosides first formed on Earth, but at some point between the formation of a stable hydrosphere 4.4 billion years ago (4.4 Ga)^1^ and the emergence of life’s last universal common ancestor^2^, RNA (and DNA) must have formed and been harnessed by life.

The limited evidence available for Earth’s earliest history allows only broad-brush geochemical pictures to be considered. However, any discussion of the emergence of nucleosides on Earth must be tempered by consideration of what is prebiotically plausible. There is no definitive catalogue of (geo)chemical reactions that would have occurred on the Earth before the advent of life, and, even if there were, it is likely that most abiotic reactions would not play a significant role in initiating life. Clearly, some restrictions to standard organic chemistry must be made for a reaction to be considered prebiotically plausible. Orgel proposed that for a reaction run in a laboratory to qualify as prebiotic it must:

- use starting materials that could have been present in adequate amounts;
- proceed in water or without solvent;
- form the relevant product in a significant yield^3^.

This flexible definition allows a broad range of chemical reactions to be investigated without losing sight of the end goal—discovering the reactions that led to life. Although liberal, these constraints are informative and, relative to modern synthetic practices, highly restrictive. Limiting chemical reactions to those that proceed in water is geochemically reasonable, as water is the most abundant solvent on the Earth (5/7th of the Earth’s surface is water-covered). However, chemically this limitation is massively restrictive and extremely challenging due to the low solubility of many organic molecules in water, the limited range of acidity and basicity available in water compared to organic solvents, as well as the potential for water itself to react as a competing nucleophile. On the other hand, the essential biochemical components which life uses are predisposed to function in an aqueous environment. Therefore, the key question must be how these components were first assembled and recruited into biology.

Hadean geochemistry can be used inform the selection of likely starting materials and environments, although caution must be exercised because models of this ancient aeon are constantly changing. For example, ideas about the composition of the early Earth’s atmosphere have radically changed over the last 70 years. The Hadean atmosphere was certainly different from the modern atmosphere: molecular oxygen, for example, is predominantly biogenic on Earth and would not have been present in significant quantities. The Jack Hills zircons provide evidence for liquid water on Earth 4.4 Ga^1^, placing rough constraints on global surface temperatures and, when coupled with predicted solar flux, the atmospheric composition. Accordingly, it has been suggested that the partial pressure of greenhouse gases must have compensated for the lower intensity of solar radiation to maintain liquid water on the early Earth, but the precise composition is open to debate^4^. Initially, a reducing atmosphere with substantial quantities of CH_4_ was thought to be likely, but this is currently deemed unrealistic due to the rapid destruction of CH_4_ by UV light^5^, and an atmosphere that contained mostly N_2_ and CO_2_ is considered more likely. Experimentally, it has been shown that energetic input (e.g. UV light or electric discharge) into mixtures of N_2_, H_2_O, H_2_ and either CH_4_ or CO_2_ produces highly complex mixtures of organic molecules, a very small fraction of which could be considered biologically relevant^6^. Similarly complex mixtures of molecules have been found on carbonaceous meteorites^7^, which suggests that complexity is almost inevitable when enough energy is supplied to simple carbon-containing systems. A more difficult, and likely more important, question is how the prebiotic molecules that are useful to life could be selected from amongst the other, chemically similar, compounds. Improved selectivity in prebiotic reaction pathways can certainly provide part of the answer; physical processes, such as selective crystallisation^8^, must also have been important.

Although the environment from which life first emerged is far from certain, determining the key prebiotic environment(s) that can support the synthesis, purification and accumulation of the essential building blocks of biology can, and should, be a two-way process between mechanistic chemistry and planetary science. Chemical constraints can be used to ascertain the likely geochemical environments of interest. For example, reactions between alcohols and phosphate salts (necessary for the formation of nucleotides) are prohibited in aqueous solution but are facile under dry conditions^3^; as such, these chemical constraints suggest that environments that can support wet-dry cycles (e.g. small ponds) could have been essential during prebiotic nucleotide synthesis. Cross-referencing these chemically constrained conditions with planetary scenarios can further constrain the potential environments or open new avenues of geochemically informed synthesis. However, ultimately all such scenarios could be pre-emptively restrictive until we have more evidence to implicate a particular chemical pathway. The Earth’s geochemistry is hugely heterogeneous, with great regional variations across the planet. Locally high concentrations of rare minerals may have been present, particularly given the increased prevalence of meteorite impacts during the Hadean aeon. Stringent restrictions upon which minerals and geochemicals would or would not have been abundant are not necessarily helpful to the question at hand given the huge uncertainty in current models, limited direct evidence of Hadean geochemistry and the unknown role of niche environments during the origin of life.

**Fig. 1:**
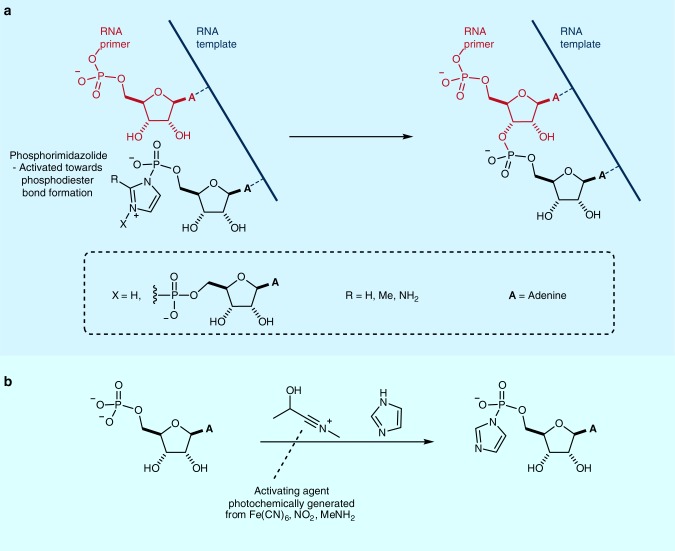
Phosphorimidazolide activation of nucleotides. **a** Phosphorimidazolide mediated non-enzymatic template-directed RNA primer extension. **b** Prebiotically plausible synthesis of adenosine-5′-phosphorimidazolide (R = H), using a photochemically generated activating agent

The uncertainty about the exact conditions required at the origin of life should not be discouraging, nor should it be used to prohibit research. Over time, our models have become more detailed and they, and our concept of what is prebiotic, will continue to evolve. For example, despite the potential importance of trace chemical species in the origins of life, as feedstocks or catalysts, it is difficult to determine their abundance on the early Earth. The rarity of trace species inevitably results in their limited impact on the already scarce rock record. Further work modelling trace species is required. However, importantly, Kepler data suggest that approximately 1-in-5 stars host rocky planets in their habitable zone^9^, and characterising their atmospheres may provide an unrivalled experimental window on the atmospheric composition of early Earth-like planets that can be used to sharpen our models of prebiotic plausibility further. Nevertheless, defining a prescriptive list of chemicals currently thought to have been available on the early Earth would likely be more restrictive than helpful in the long term, as any such list will inevitably change over time. A potentially illuminating case study, that highlights such a change, is the use of phosphorimidazolides as substrates in studies of non-enzymatic RNA replication (Fig. 1a)^10,11^.

**Fig. 2:**
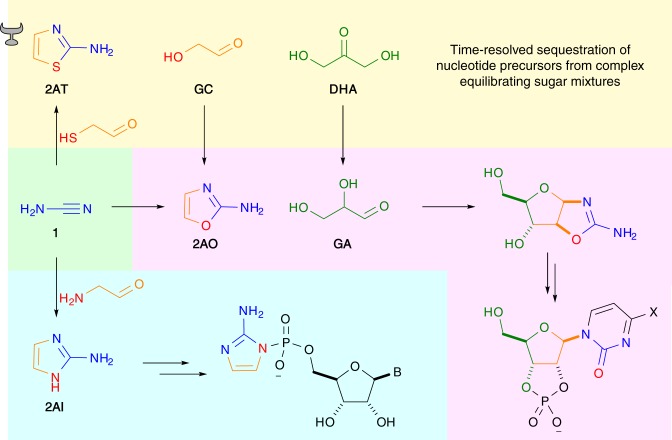
The roles of 2-aminoazoles in prebiotically plausible nucleotide chemistry. Green: Divergent synthesis of 2-aminothiazole (**2AT**), 2-aminooxazole (**2AO**) and 2-aminoimidazole (**2AT**) from cyanamide (**1**). Yellow: Crystallisation-controlled aldehyde sequestration by 2-aminothiazole (**2AT**) giving access to pure glycolaldehyde (**GC**) and glyceraldehyde (**GA**), in the order required for selective nucleotide synthesis, from equilibrating sugar mixtures. Pink: Prebiotically plausible pyrimidine nucleotide synthesis. Cytidine X = NH_2_, uridine X = OH. Blue: Nucleoside-5′-phosphorimidazolide activation with 2-aminoimidazole (**2AI**). B = nucleobase

These activated nucleotides react with a short strand of RNA (a primer, bound to templating complementary RNA) much more facilely than (biological) nucleoside triphosphates. Researchers studying phosphorimidazolides have typically presented them as useful models to probe the difficult problem of non-enzymatic RNA replication, rather than as prebiotically plausible substrates. However, imidazolide studies have provided great mechanistic insights into abiotic nucleic acid replication over the past 50 years, and this has ultimately prompted the discovery of a prebiotically plausible route for their formation (Fig. 1b)^12^. Recently, these studies also led to the discovery that, out of many different imidazole-type molecules, 2-aminoimidazole (**2AI**) is the superior activating agent^13^. Interestingly, **2AI** can be made through a prebiotically plausible reaction which is remarkably similar to the synthesis of two other molecules that have been implicated in prebiotic nucleotide chemistry: 2-aminooxazole (**2AO**) and 2-aminothiazole (**2AT**) (Fig. 2). **2AO** provides a robust and selective route to nucleotides which bypasses free ribose (Fig. 2, pink)^14,15^, while **2AT** is able to chaperone this synthesis via selective time-resolved crystallisation of the required aldehydes, **GC** and **GA**, from highly complex sugar mixtures, and dynamically resolving C3 aldose sugar **GA** from its more thermodynamically stable ketose isomer **DHA** (Fig. 2, yellow)^8^. Structurally, these three azole molecules are remarkably similar and could all share a common origin from cyanamide (**1**). They play three distinct roles in facilitating prebiotically plausible nucleotide chemistry, but none of these would have been considered prebiotic molecules 15 years ago. It is possible that these imidazolide, and the prebiotically plausible pathways they implicate, will be superseded by more efficient, more robust and more accurate prebiotic pathways to RNA, but by studying them invaluable information about the chemical reactivity of simple aqueous systems that can selectively build nucleic acids has been discovered. Had researchers ignored (or been dissuaded from investigating) these systems on the grounds that they were thought not to be prebiotically plausible, these insights may have been missed.

Ultimately, until several routes have been found by which simple abiotic molecules can be transformed into systems on which Darwinian evolution can act, discussions about which reactions are more (or less) prebiotically plausible than others are simply less instructive than investigating chemical reactivity. By limiting the scope of our investigations too strictly, we will always risk missing crucial aspects of the story as it unfolds.
